# Mangiferin Alleviates Renal Interstitial Fibrosis in Streptozotocin-Induced Diabetic Mice through Regulating the PTEN/PI3K/Akt Signaling Pathway

**DOI:** 10.1155/2020/9481720

**Published:** 2020-01-31

**Authors:** Yanyan Song, Wei Liu, Ke Tang, Junting Zang, Dong Li, Hang Gao

**Affiliations:** ^1^Department of Nephrology, The Second Hospital of Jilin University, No. 218 Ziqiang Street, Changchun 130021, China; ^2^Department of Spinal Surgery, The First Hospital of Jilin University, No. 71 Xinmin Street, Changchun, Jilin 130021, China; ^3^Department of Electrical Diagnosis, The Second Hospital of Jilin University, No. 218 Ziqiang Street, Changchun 130021, China; ^4^Department of Bone and Joint Surgery, The First Hospital of Jilin University, No. 71 Xinmin Street, Changchun 130021, China; ^5^Department of Immunology, College of Basic Medical Sciences, Jilin University, No. 126 Xinmin Avenue, Changchun, Jilin Province 130021, China

## Abstract

Renal interstitial fibrosis is considered to be the typical manifestation of diabetic nephropathy (DN). Mangiferin has shown positive effect on the prevention or treatment of diabetes and its complications. The aim of this study was to explore the inhibitive effect and mechanism of mangiferin on renal interstitial fibrosis in diabetic mice. Streptozotocin- (STZ-) induced diabetic mice were treated with mangiferin (15, 30, and 60 mg/kg/d) for 4 weeks. The morphology of kidneys was observed by Masson's trichrome staining, and the biochemical parameters (fasting blood glucose (FBG), triglyceride (TG), total cholesterol (TC), blood urea nitrogen (BUN), serum creatinine (SCr), and urine protein) were determined by kits. In addition, the levels of inflammatory cytokines (tumor necrosis factor-*α* (TNF-*α*), interleukin- (IL-) 6, and IL-1*β*), antioxidant enzymes (SOD, CAT, and GSH-Px), MDA, and ROS were assessed. Furthermore, the expressions of fibronectin (FN), collagen I (Col I), and *α*-SMA were measured by immunohistochemistry. Regulations of TGF-*β*1 and the PTEN/PI3K/Akt pathway were detected by Western blotting. Treatment with mangiferin significantly ameliorated renal dysfunction in diabetic mice, as evidenced by the increase in body weight and decreases in FBG, TG, TC, BUN, SCr, urine protein, and the kidney to body weight ratio (KW/BW). Furthermore, mangiferin treatment prevented renal interstitial fibrosis evidenced by decreases in the positive expression of FN, Col I, and *α*-SMA, in comparison with morphological changes in the renal tissue. Meanwhile, mangiferin increased antioxidant enzymes, reduced the TNF-*α*, IL-6, and IL-1*β*, as well as MDA and ROS. Additionally, mangiferin administration also downregulated TGF-*β*1, upregulated PTEN, and decreased the phosphorylation of both PI3K and Akt. These findings demonstrate that mangiferin may reduce inflammation and oxidative stress in DN, thereby inhibiting the renal interstitial fibrosis by reducing the TGF-*β*1-mediated elevation of Col I, FN, and *α*-SMA through the PTEN/PI3K/Akt pathway.

## 1. Introduction

Diabetes is a kind of metabolic disease characterized by chronic elevated blood glucose levels. Hyperglycemia is caused by the impaired production of insulin and resistance of insulin [1]. Long-term chronic hyperglycemia induces disorders of fat and protein metabolism, which then causes a series of complications in retinal, kidney, and nerve systems [2–4]. Diabetic nephropathy (DN) is the most serious complication of diabetes. It is characterized by the loss of renal cells and their replacement by extracellular matrix (ECM), eventually leading to glomerulosclerosis and tubulointerstitial fibrosis [5].

Oxidative stress and chronic inflammation play crucial roles in the development of DN [6, 7]. Hyperglycemia increases the generation of reactive oxygen species (ROS), which activate signal transduction and induce the increases of fibrotic factors, such as fibronectin (FN), *α*-smooth muscle actin (*α*-SMA), and collagen I [3, 8]. Furthermore, ROS activate the expression of transforming growth factor- (TGF-) *β*1, which contributes to the accumulation of ECM as a key characteristic in renal fibrosis [3, 9]. Scientific evidence also suggests the inflammatory factors, tumor necrosis factor-*α* (TNF-*α*) and interleukin-6 (IL-6), are well known to be associated with the development of renal disease in diabetes [10]. In addition, the phosphoinositide 3-kinase (PI3K)/protein kinase B (Akt) signaling pathway has been demonstrated to play a critical role in proliferation, progression of cell cycle, and cell viability in diabetes [11]. Phosphatase and tensin homologue deleted on chromosome 10 (PTEN) is a negative regulator of the PI3K signaling; it can inhibit the activation of Akt. A significant decrease in the PI3K and Akt was observed in streptozotocin- (STZ-) induced DN of rats [12]. Therefore, antioxidative stress and anti-inflammation are essential approaches for the prevention and treatment of renal fibrosis in DN. It is necessary and urgent to find natural, effective, and safe drugs to treat DN.

Mangiferin (2-*β*-D-glucopyranosyl-1,3,6,7-tetrahydroxy-9H-xanthen-9-one) is a major active ingredient in the rhizomes of *Anemarrhena asphodeloides* Bunge, a well-known traditional Chinese medicine [13]. Mangiferin possesses several beneficial biological activities such as antioxidant, antimicrobial, antidiabetic, antiallergic, anticancer, hypocholesterolemic, and immunomodulatory [14, 15]. The reports suggest that mangiferin has a positive effect on the prevention or treatment of diabetes and its complications. Although the beneficial effects of mangiferin on DN have also been confirmed in previous studies, reports regarding the mechanisms of mangiferin on renal interstitial fibrosis in DN are limited.

In this study, STZ-induced diabetic mice were used as models to study the protective effect of mangiferin on diabetic renal interstitial fibrosis injury and to explore the mechanism of the PTEN/PI3K/Akt signaling pathway in mangiferin inhibiting renal interstitial fibrosis in DN, which might be able to provide more theoretical evidence for clinical application of traditional Chinese medicine on treatment of diabetes.

## 2. Materials and Methods

### 2.1. Mice

A total of 70 C57BL/6 male mice (7 weeks old) weighing 21 g ± 2 g were obtained from the Experimental Animal Center at Jilin University (Jilin, China). The experiments had been approved by the ethics committee of the Second Hospital of Jilin University. All animal experiments were performed in accordance with the National Guidelines for Experimental Animal Welfare and with approval of the Animal Welfare and Research Ethics Committee at Jilin University (Changchun, China). The mice were housed in the SPF condition with constant 22 to 25°C room temperature, 45-55% humidity, a 12-hour light-dark cycle, and accessible clean food and water *ad libitum*.

The diabetic mice models were performed as described with some modifications [3]. After acclimatization for one week, mice were divided into two groups; the mice in the control group (Con, *N* = 10) were treated with citric acid buffer, whereas the model mice (*N* = 60) were given injection with multiple low-dose STZ (50 mg/kg, Sigma Aldrich, St. Louis, MO, USA). Injections were repeated in 5 consecutive days. STZ was dissolved in 0.1 mol/L ice-cold citric acid buffer (pH 4.5), and the injection was completed within 30 min. Mice with fasting blood glucose (FBG) higher than 13.9 mmol/L (250 mg/dL) after 72 h were established as successful diabetes model mice. Mangiferin (>97% purity, China Food and Drug Regulatory Research Institute, Beijing, China) was suspended in distilled water and was given to the diabetic mouse by oral gavage once daily. Bisperoxovanadium (BpV, HOpic) (Selleck Chemicals, USA) is a highly potent inhibitor of PTEN with an IC_50_ of 14 nM. Diabetic mice were divided into 6 groups randomly (*N* = 10): model group (Mod), mangiferin in low dose group (Mang-L, 15 mg/kg/d), mangiferin in middle dose group (Mang-M, 30 mg/kg/d), mangiferin in high dose group (Mang-H, 60 mg/kg/d), PTEN inhibitor group (BpV, diabetic mice were injected with PTEN inhibitor and given normal saline), and PTEN inhibitor+Mangiferin group (BpV+Mang-H, diabetic mice were injected with PTEN inhibitor and given mangiferin 60 mg/kg/d).

### 2.2. Assessment of Biochemical Parameters

The body weights (BW) of the mice were weighted before sacrificed. The mice were sacrificed by anesthetizing with ketamine (30 mg/kg) and thiobutabarbital (50 mg/kg) after experimental 4 weeks. The blood was collected in test tubes with heparin solution via the caudal vena cava, followed by serum separation. The urine was collected from the bladder to measure the urine protein. Fasting blood glucose (FBG), triglyceride (TG), total cholesterol (TC), blood urea nitrogen (BUN), serum creatinine (SCr), and urine protein in the urine and serum were measured according to the manufacturer's protocol for each kit (Jiancheng Bioengineering Institute, Nanjing, China). The kidneys were collected and weighted to calculate the kidney to body weight ratio (KW/BW). The samples were stored at -80°C for further analysis.

### 2.3. Histological Analysis

Masson's trichrome staining was performed as described before [13] with some modifications. The kidney tissue was fixed in 10% formalin, and routinely paraffin-embedded, 4 *μ*m thick sections of the sample were dewaxed, hydrated, and then stained with Masson's trichrome, cleared in xylene, and mounted with neutral balsam. The stained sections were examined under a light microscope (Olympus BX-50, Japan) at 400x magnification by two blinded pathologists. Three points in the central sections of each lesion were analyzed, expressed as the approximate proportion of stained collagen fibers to the total stained part. Image Pro Plus 6.0 was used to analyze the optical density (OD) of image results.

### 2.4. Measurement of ROS

The kidney tissue was homogenized with saline (1 : 9 *w*/*v*) at 4°C. The homogenates were centrifuged at 11000g for 15 min at room temperature. ROS production was measured by using a 2,7-dichlorofluorescein diacetate (DCFH-DA) kit (Beyotime Biotech Inc., Shanghai, China). The fluorescence was collected by a microplate reader (BioTek Instruments, USA) equipped with a fluorescence detector (the excitation wavelength was 488 nm, and emission wavelength was 510 nm).

### 2.5. Measurement of MDA and Antioxidant Enzymes

The kidney tissue was homogenized, and protein concentration was measured using a BCA protein assay kit (Beyotime Biotech Inc., Shanghai, China). Malondialdehyde (MDA) content, levels of superoxide dismutase (SOD), catalase (CAT), and glutathione peroxidase (GSH-Px) were measured using commercial kits (Beyotime Biotech Inc., Shanghai, China) according to the manufacturer's instructions.

### 2.6. Analysis of Inflammatory Factors in Kidney Tissues

The interleukin-6 (IL-6, No. PI330), interleukin-1*β* (IL-1*β*, No. PI305), and tumor necrosis factor-*α* (TNF-*α*, No. PT518) concentrations in the renal homogenates were measured using commercial ELISA kits (Beyotime Biotech Inc., Shanghai, China) according to the manufacturer's instructions.

### 2.7. Immunofluorescence (IF) Staining

Immunofluorescence staining was performed as described before [16] with some modification, to detect the expression levels and differences of fibronectin (FN), collagen I (Col I), and *α*-smooth muscle actin (*α*-SMA) in the kidneys of five groups (Mod group, Mang-H group, BpV group, and BpV+Mang-H group). The sections of the paraffin-embedded sample were made and blocked with serum-free protein (Dako, Victoria, Australia) and permeabilized for 30 min. Sections were incubated with primary antibodies FN, Col I, *α*-SMA (Wanleibio, Shijiazhuang, China; 1 : 100 dilution) overnight at 4°C. Sections were washed with TBS 3× 10 min and incubated with species-specific secondary antibodies: Goat Anti-Rabbit IgG H&L (Cy3®) (1 : 1000 dilution, ab6939, Abcam, USA) at room temperature for 1 h. After washing with TBS, the sections were stained with Glycerol Mounting Medium (Abcam) that contained 4,6-diamidino-2-phenylindole (DAPI) and 1,4-diazobicyclo-2,2,2-octane (DABCO). Labelled tissues were visualized using a Leica DM LB2 microscope. Fluorescence images (400x magnification) were captured using NIS-Elements 4.13 (Nikon, Japan) software. The number of fluorescence-positive cells was counted from five representative high-power fields (400x magnification) per tissue section.

### 2.8. Western Blot

Total protein concentration of homogenate was measured using a BCA protein assay kit (Beyotime Biotech Inc., Shanghai, China) according to the manufacturer's protocol and was equalized before electrophoresis. Briefly, 40 *μ*g of the proteins in the supernatant was separated by 10% SDS-PAGE and transferred onto PVDF membranes. After blocking at room temperature for 1 h with 5% nonfat dry milk, the membranes incubated with antibodies TGF-*β*1 (1 : 500 dilution, Wanleibio, Shijiazhuang, China), PTEN (1 : 400 dilution, Wanleibio, Shijiazhuang, China), PI3K p85 (1 : 1000 dilution, Cell Signaling Technology, USA), p-PI3K p85 (Tyr458)/p55 (Tyr199) (1 : 1000 dilution, Cell Signaling Technology, USA), and Akt and p-Akt (Ser473) (1 : 1000 dilution, Cell Signaling Technology, USA) overnight at 4°C. After washing with TBST, the membranes were incubated with IgG-HRP (1 : 5000 dilution, Wanleibio, Shijiazhuang, China) for 1 h at room temperature. The membranes were developed with enhanced chemiluminescence using ECL reagents (Beyotime Biotech Inc., Shanghai, China) and visualized using a digital imaging system (Bio-Rad Laboratories, Inc., USA). The blots were quantitated by densitometric analysis using NIH ImageJ software. The data were normalized on the basis of GAPDH level.

### 2.9. Statistical Analysis

Data were expressed as mean ± standard error of mean (SEM). Differences between groups were assessed with one-way analysis of variance (ANOVA), followed by Tukey's test using the SPSS 21.0 statistical package (IBM Corp., Armonk, NY). Differences were considered statistically significant at *P* < 0.05.

## 3. Results

### 3.1. Mangiferin Reduces FBG and Elevates Body Weight of STZ-Induced Diabetic Mice

**Figure 1:**
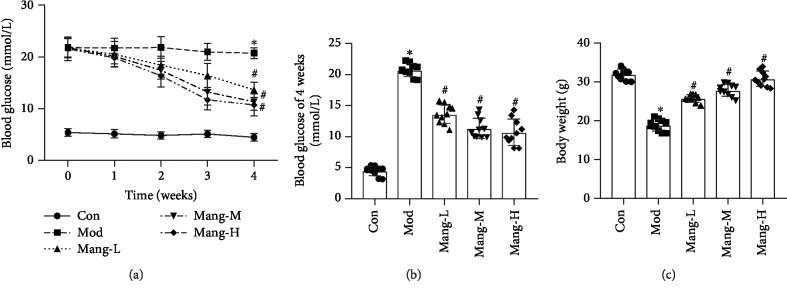
Effects of mangiferin on fasting blood glucose (FBG) and body weight of diabetic mice. (a) FBG of weeks 1, 2, 3, and 4. (b) FBG of week 4. (c) Body weight. Data are expressed as the mean ± S.D., *N* = 10, ^∗^*P* < 0.05 versus the Con group, ^#^*P* < 0.05 versus the Mod group.

As shown in Figures 1(a) and 1(b), FBG was found to be significantly elevated in STZ-induced diabetic mice as compared to normal mice (*P*<0.05). At weeks 1, 2, 3, and 4, treatment with mangiferin (15, 30, and 60 mg/kg/d) resulted in a concentration-dependent reduction in FBG as compared to model mice (*P*<0.05), but not completely reduced to the level of normal mice (Figures 1(a) and 1(b)). The body weights of model mice were significantly decreased compared with the normal mice. After treated with mangiferin, the body weights were increased in a dose-dependent manner compared with the model mice (*P* < 0.05, Figure 1(c)). These results indicate that mangiferin exhibits antidiabetic effect on STZ-induced diabetic mice.

### 3.2. Mangiferin Alleviates Kidney Dysfunction and Lipid Metabolism of Diabetic Mice

**Figure 2:**
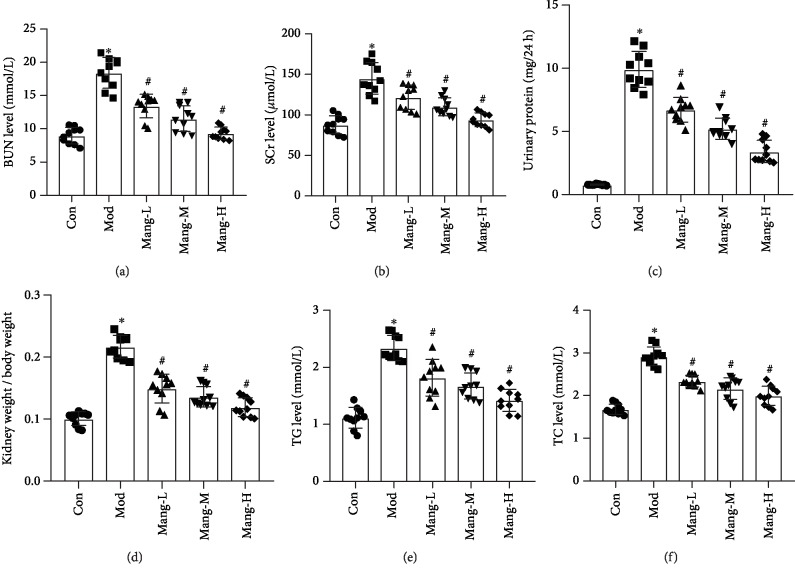
Effects of mangiferin on biochemical parameters of kidney dysfunction and lipid metabolism in diabetic mice. (a) Blood urea nitrogen (BUN) level. (b) Serum creatinine (SCr) level. (c) Urine protein level. (d) Kidney to body weight ratio (KW/BW). (e) Triglyceride (TG) level. (f) Total cholesterol (TC) level. Data are expressed as the mean ± S.D., *N* = 10, ^∗^*P* < 0.05 versus the Con group, ^#^*P* < 0.05 versus the Mod group.

Specific markers related to kidney dysfunction such as BUN, SCr, and urine protein, as well as the kidney to weight ratio (KW/BW), were shown in Figure 2. The significant elevations of BUN, SCr, and urine protein were observed in model mice (*P* < 0.05, Figures 2(a)–2(c)). However, mangiferin treatment efficiently reduced the elevations of these biochemical parameters. In addition, the KW/BW of diabetic mice also reduced by mangiferin compared with the model mice (*P* < 0.05, Figure 2(d)). Furthermore, treatment with mangiferin significantly reduced the levels of TG and TC in dose-dependent manners, which were elevated in the model mice (*P* < 0.05, Figures 2(e) and 2(f)). These results indicate that mangiferin exhibits protective effects on renal injuries and lipid metabolism disorders in STZ-induced diabetic mice.

### 3.3. Mangiferin Protects against the Renal Injury of STZ-Induced Diabetic Mice

**Figure 3:**
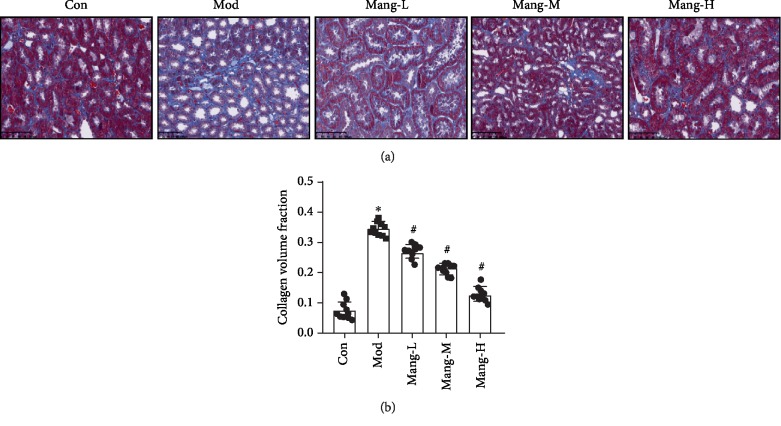
The histopathology of mangiferin effects on renal injury in STZ-induced diabetic mice. (a) Masson's trichrome staining, ×400 (Scale bar = 50 *μ*m). (b) Semiquantitative analysis of stained positive area in the lesions. Data are expressed as the mean ± S.D., *N* = 10, ^∗^*P* < 0.05 versus the Con group, ^#^*P* < 0.05 versus the Mod group.

Masson's trichrome staining was used to observe the effects of mangiferin on the renal fibrosis of diabetic mice. The collagen fiber was stained blue, and the muscle fiber cytoplasm was stained red. These changes in the glomerulus of the control and experimental groups are shown in Figure 3. A little renal collagen fiber deposition was observed in the untreated mice (Con group). But in the Mod group, the basement membrane of tubules was thickened, and renal interstitial fibrosis was observed. A significant increase of collagen volume was observed in the tubulointerstitium of mice treated with STZ alone (Mod group, *P* < 0.05, Figure 3(b)). These changes of mesenchymal collagen fiber were effectively alleviated with mangiferin treatment (Mang-L, Mang-M, and Mang-H) with dose-dependent manners (*P* < 0.05, Figure 3(b)). Especially in high-dose-treated mice, the visible collagen fibers were quite near to that of the control group. These results further show that mangiferin exhibit renal protective effect on DN.

### 3.4. Mangiferin Reduces the Elevated Levels of Inflammatory Factors in Diabetic Mice

**Figure 4:**
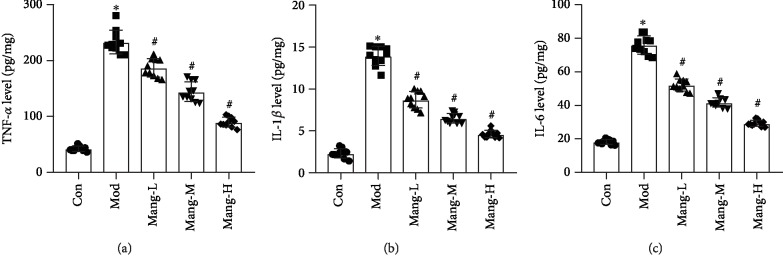
Effects of mangiferin on inflammation in the kidney of STZ-induced diabetic mice. (a) Tumor necrosis factor-*α* (TNF-*α*) level. (b) Interleukin-1*β* (IL-1*β*) level. (c) Interleukin-6 (IL-6) level. Data are expressed as the mean ± S.D., *N* = 10, ^∗^*P* < 0.05 versus the Con group, ^#^*P* < 0.05 versus the Mod group.

The TNF-*α* level increased in the model group, while the administration of mangiferin was found to decrease the level of TNF-*α* in kidney in a dose-dependent manner (*P* < 0.05, Figure 4(a)). In addition, treatment with mangiferin also decreased the levels of IL-1*β* and IL-6 in dose-dependent manners compared to the model group, which were significantly increased in diabetic mice in comparison with the normal mice (*P* < 0.05, Figures 4(b) and 4(c)). These results suggest that the protective effect of mangiferin on diabetic renal injury is partly due to its anti-inflammatory effect.

### 3.5. Mangiferin Plays an Antioxidant Role in STZ-Induced Diabetic Mice

**Figure 5:**
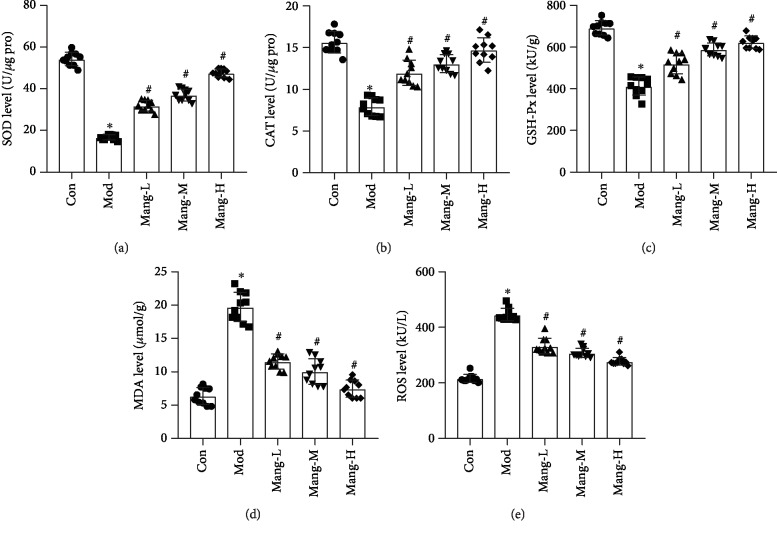
Effects of mangiferin on antioxidant activities in the kidney of STZ-induced diabetic mice. (a) Superoxide dismutase (SOD) level. (b) Catalase (CAT) level. (c) Glutathione peroxidase (GSH-Px) level. (d) Malondialdehyde (MDA) level. (e) Reactive oxygen species (ROS). Data are expressed as the mean ± S.D., *N* = 10, ^∗^*P* < 0.05 versus the Con group, ^#^*P* < 0.05 versus the Mod group.

As shown in Figure 5, the antioxidant enzyme activities (SOD, CAT, and GSH-Px) were significantly decreased in model mice, along with the significant elevation of MDA and ROS levels (*P* < 0.05), while treatment with mangiferin increased the activities of SOD, CAT, and GSH-Px in a dose-dependent manner (*P* < 0.05, Figures 5(a)–5(c)). Also, the decreasing MDA and ROS levels were found with administration of mangiferin in a dose-dependent manner (*P* < 0.05, Figures 5(d) and 5(e)). These results suggest that the protective effect of mangiferin on diabetic renal injury partly results from its antioxidative stress effect.

### 3.6. Mangiferin Alleviates the Renal Fibrosis of STZ-Induced Diabetic Mice

**Figure 6:**
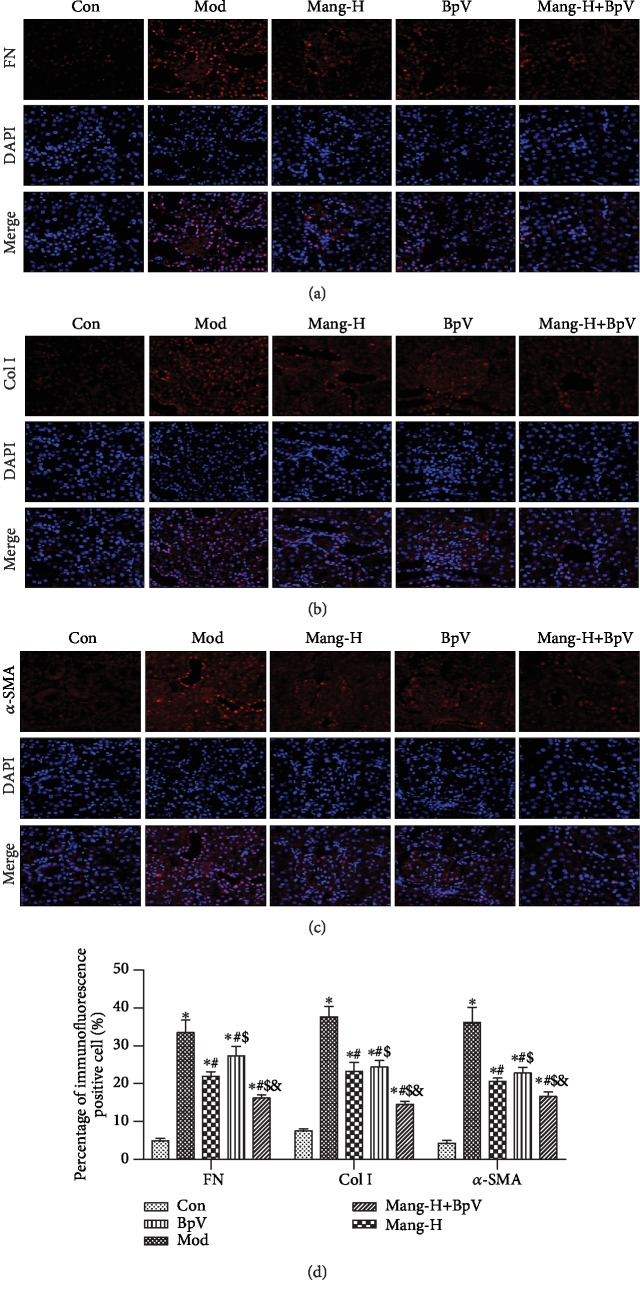
Immunofluorescence analysis for the effect of mangiferin on expressions of fibronectin (FN), collagen I (Col I), and *α*-smooth muscle actin (*α*-SMA) in diabetic mice. (a) Immunofluorescence of fibronectin (FN). (b) Immunofluorescence of collagen I (Col I). (c) Immunofluorescence of *α*-smooth muscle actin (*α*-SMA). (d) Quantitative results. ^∗^*P* < 0.05 versus the Con group, ^#^*P* < 0.05 versus the Mod group, ^$^*P* < 0.05 versus the Mang-H group, ^&^*P* < 0.05 versus the BpV group. ns: no significance.

To further investigate the protective effect of mangiferin on the renal fibrosis of diabetic mice, the expressions levels and differences of ECM-related factors, such as fibronectin (FN), collagen I (Col I), and *α*-SMA in the renal cortex, were observed in Figure 6. Compared with the normal group, the expressions of FN, Col I, and *α*-SMA were significantly increased in kidneys of diabetic mice (*P* < 0.05). Nevertheless, treatment with high-dose mangiferin effectively reversed the elevation of FN, Col I, and *α*-SMA (*P* < 0.05). Additionally, with the use of the PTEN inhibitor (BpV), it was found that the expressions of FN, Col I, and *α*-SMA were significantly higher than those of model mice (*P* < 0.05). However, mangiferin treatment also exhibited significant decreases of FN, Col I, and *α*-SMA levels in the BpV-treated diabetic mice (*P* < 0.05) but did not surpass the mangiferin treatment group without PTEN inhibitor. These results suggest that the protective effect of mangiferin on renal interstitial fibrosis is related to its inhibitory effect on generation and accumulation of ECM factors.

### 3.7. Mangiferin Regulated TGF-*β*1 and the PTEN/PI3K/Akt Signaling Pathway

**Figure 7:**
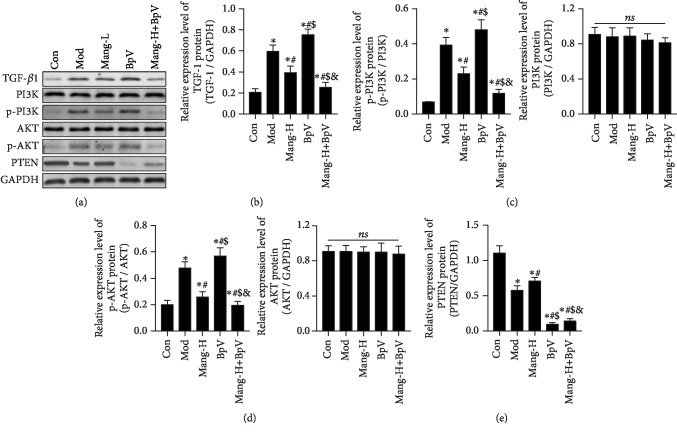
Effects of mangiferin on TGF-*β*1 and the PTEN/PI3K/Akt signaling pathway. (a) Western blot band. (b) The protein expressions of TGF-*β*1. (c) The protein expressions of p-PI3K p85 (Tyr458)/p55 (Tyr199) and PI3K p85. (d) The protein expressions of p-Akt (Ser473) and Akt. (e) The protein expressions of PTEN. Data are expressed as the mean ± S.D., *N* = 4, ^∗^*P* < 0.05 versus the Con group, ^#^*P* < 0.05 versus the Mod group, ^$^*P* < 0.05 versus the Mang-H group, ^&^*P* < 0.05 versus the BpV group. ns: no significance.

In order to be more definitive, the related signaling pathways that regulate ECM were evaluated. Figures 7(a) and 7(b) show the inhibitive effect of mangiferin on the upstream TGF-*β*1 signaling. Compared with the normal group, the expression of TGF-*β*1 was significantly increased in kidneys of diabetic mice (*P* < 0.05), while treatment with high-dose mangiferin effectively reversed the elevation of TGF-*β*1 (*P* < 0.05), which was consistent with the expressions of FN, Col I, and *α*-SMA close to the normal level. Additionally, with the intervention of BpV, expression of TGF-*β*1 was significantly higher than that of model mice (*P* < 0.05). Nevertheless, compared with the BpV group, mangiferin treatment significantly decreased the TGF-*β*1 expression in the BpV-treated diabetic mice (*P* < 0.05). These results suggest that the protective mechanism of mangiferin on renal interstitial fibrosis in part associates with the inhibitory of TGF-*β*1-mediated ECM elevation.

The inhibitive effect of mangiferin on the PTEN/PI3K/Akt signaling pathway was shown in Figures 7(a) and 7(c)–7(e). In the model group, the protein expressions of p-PI3K/PI3K and p-Akt/Akt were significantly higher than those in the normal group (*P* < 0.05); on the contrary, the expression of PTEN was significantly decreased (*P* < 0.05). With the intervention of BpV, expressions of p-PI3K/PI3K and p-Akt/Akt were further increased compared to model mice (*P* < 0.05). However, compared with the BpV group, mangiferin treatment significantly decreased the p-PI3K/PI3K and p-Akt/Akt expressions and simultaneously increased PTEN proteins in the BpV-treated diabetic mice (*P* < 0.05). These findings demonstrate that mangiferin increases resistance to renal interstitial fibrosis by modulating the PTEN/PI3K/Akt signaling pathway, which can serve not only as a noninvasive biomarker but also as a pathologic mediator and therapeutic target of kidney fibrosis.

## 4. Discussion

Diabetic nephropathy (DN) is one of the most severe diabetic complications. Renal fibrosis, especially glomerulosclerosis and tubulointerstitial fibrosis, is considered to be the final manifestations of DN [17]. In order to develop effective preventable and therapeutic strategies of DN, it is essential to understand the cellular and molecular mechanisms underlying fibrotic development. Mangiferin is a xanthone present in *Anemarrhena asphodeloides* Bunge. It exhibits tremendous health-related properties such as antiviral, anticancer, antidiabetic, antioxidative, antiaging, immunomodulatory, hepatoprotective, and analgesic effects. In previous studies, mangiferin exhibited beneficial effects on diabetes and diabetic complications, especially focused on DN [13, 18, 19]. The current study investigated the effects of mangiferin on the renal fibrosis of diabetic mice and expounded the underlying mechanisms associated with the PTEN/PI3K/Akt signaling pathway. The present study suggested that the potential abilities of mangiferin on DN in vivo were related to the inhibitory effect of renal interstitial fibrosis.

STZ is usually used to establish the animal diabetic model; one of the characteristics in pathology is inducing diabetic renal changes [20, 21]. Thus, a continued low dose of STZ-induced diabetic mice was selected as model for investigating the effect of mangiferin on renal injury. Elevated glucose levels successfully induced renal lesions that were similar with human patients of DN, which is characterized by hyperglycemia, hyperlipidemia, oxidative stress, and renal damage [22]. In the present study, STZ-induced diabetic mice showed development of renal injury by histomorphological and biochemical analysis, which was significantly ameliorated by mangiferin treatment. The results demonstrated that mangiferin could decrease the FBG levels and increase KW/BW ratios of diabetic mice. A previous study also revealed that mangiferin reduced the plasma glucose level and restored kidney to body weight ratio [7]. The biochemical levels including SCr and BUN are critical indicators of DN which gradually increased in diabetic patients and in turn further accelerated the development of DN [23, 24]. In this study, mangiferin significantly decreased SCr, BUN, and urine protein in STZ-induced diabetic mice, which meant the mangiferin could partially mitigate the progression of DN. These further confirmed the beneficial effects of mangiferin on diabetes, focused on glycemic control and renal protection. The improved effect of mangiferin on renal fibrosis may also be related to regulating FBG level, which also contributes to its effects that we cannot ignore. However, in this study, although mangiferin can reduce FBG, it did not reduce to the normal level of control. From this, it can be speculated that the improvement of mangiferin on renal fibrosis is not completely determined by the inhibitory effect of glycemia.

Abnormal lipid metabolism often occurs in the pathogenesis of DN [25]. As the primary lipid metabolic factors, TC and TG, are typically increased in diabetic patients [26]. In the study, TC and TG levels were significantly decreased after mangiferin treatment in the STZ-induced diabetic mice, indicating that mangiferin can benefit diabetic mice via the regulation of abnormal lipid metabolism and dyslipidemia. These results are consistent with the relevant research about inhibitory effect of timosaponin B-II on elevated blood lipids in alloxan-induced mice [27]. Similarly, the flavonoids of A. asphodeloides Bunge were reported to reduce FG and TG in type 2 DM rats [28]. The antidiabetic drug metformin improves lipid metabolism and ameliorates lipid peroxidation via enhancing insulin sensitivity [3]. Thus, mangiferin might undergo a similar mechanism to regulate DN-induced dysfunction of lipid metabolism.

In the progress of DN in diabetic patients, the renal hypertrophy, nodular sclerosis, thickening of the GBM, and no proliferation of mesangial matrix are pivotal pathological characteristics [27]. In the current study, STZ-induced diabetic mice showed that remarkable fibrosis of tubulointerstitium accompanied these features, which were alleviated by mangiferin. The result demonstrated that mangiferin could markedly improve the renal injury to inhibit DN in mice. These are consistent with the previous reports [7, 17]. Relevant study also indicated that TB-II treatment ameliorated the suppression of nodular sclerosis and alleviated glomerular injuries [27]. A study demonstrated that mangiferin shows beneficial preventable and remedial effects on renal fibrosis [13]. Further, the potential mechanism was revealed.

A previous study has revealed that targeting oxidative stress and inflammation could improve therapeutic options for DN [29]. A hypothesis has been proposed that overproduction of ROS induced by high blood glucose may result in various pathogenic pathways in DN [30]. Various studies showed that mangiferin can protect kidney based on its effectively endogenous antioxidant system regulating and strong free radical (ROS) scavenging activity in diabetes [7, 17, 29]. In this study, the activities of SOD, CAT, and GSH-Px increased, accompanied by decreases of MDA and ROS levels after mangiferin treatment. These results suggested that mangiferin exerts a renoprotective effect via increasing antioxidant enzymes and reducing lipid peroxidation, which were consistent with the previous report [18].

Chronic inflammation plays an important role in the progression of DN [19]. The inflammatory cytokines, such as TNF-*α* and IL-6, are activated by the oxidative stress, PKC pathways, and chronic unresolved inflammation [31]. Previous studies have indicated that the levels of TNF-*α* and IL-6 are significantly elevated in patients with DN, as compared with hyperglycemic patients without nephropathy [32, 33]. Therefore, the anti-inflammatory drugs may serve as a supplemental strategy for the treatment of renal dysfunction in diabetic patients [34]. Mangiferin has also been shown to exert a antihypoglycemic effect by modulating glucose metabolism, ameliorating insulin resistance, lowering cholesterol synthesis, and inhibiting the expression of the TNF-*α*. In the present study, STZ markedly increased TNF-*α*, IL-1*β*, and IL-6 levels in diabetic mice, whereas the inflammatory cytokines decreased in a concentration-dependent mode by the intervention of mangiferin. Therefore, apart from antioxidative stress, the anti-inflammatory effect mediated by mangiferin is another important mechanism to protect mice from the development of DN. The outstanding effect of mangiferin on attenuating diabetic renal injury is consistent with the opinion that the anti-inflammatory and antioxidant property of mangiferin may be responsible for the alleviation of renal injuries in diabetic rats [13].

The expressions of *α*-SMA protein and collagen I and IV are increased in the fibrotic kidneys of diabetic mice [8]. Fibronectin is one of the major noncollagenous glycoproteins in the extracellular matrix and basement membrane; it plays a central role in cell adhesion, regulating cell polarity, differentiation, and growth. Previous reports have indicated that mangiferin improved renal fibrosis of diabetic animals by inhibiting glomerular ECM expansion and accumulation and decreasing glomerular basement membrane thickness and mesangial cell proliferation [18, 29]. Immunofluorescence analysis was conducted in further study to detect the change of Col I, FN, and *α*-SMA. In the results, renal fibrosis characterized by significantly elevated expression levels of Col I, FN, and *α*-SMA was detected in the model group, whereas mangiferin treatment significantly reversed the elevated protein expressions of Col I, FN, and *α*-SMA in the diabetic mice, indicating a remarkable effect of mangiferin on renal fibrosis of DN mice. A similar study also revealed that chronic mangiferin treatment exhibited good effectiveness on renal fibrosis through decreasing Col IV and *α*-SMA levels [13]. Moreover, the potential mechanisms of mangiferin effect on renal fibrosis of diabetic mice were further investigated.

TGF-*β* plays a critical role in glomerulosclerosis and interstitial fibrosis; it contributes to tissue fibrosis via stimulating ECM synthesis and reducing collagenase production [35, 36]. A variety of studies have demonstrated that TGF-*β* plays an important role in the pathogenesis of DN, which is associated with oxidative stress and inflammation [3, 35, 37]. The results have shown that the intervention of mangiferin can downregulate the protein expression of TGF-*β*1, indicating that mangiferin may inhibit TGF-*β*1 signal activation. TGF-*β*1 can enhance the deposition of ECM to initiate and promote the development of renal fibrosis [38, 39]. Therefore, this result implied that mangiferin may decrease Col I, FN, and *α*-SMA by intervening the activation of TGF-*β*1 relevant pathways, thereby alleviating the renal interstitial fibrosis in DN caused by STZ. These results are consistent with the previous research [40]. Similarly, the Ayurvedic antidiabetic medicine *Salacia oblonga* (SO) root (the major component is mangiferin) treatment reversed the increase in renal TGF-*β*1 expression in the Zucker diabetic fatty (ZDF) rat kidney [35].

Phosphoinositide 3-kinase- (PI3K-) Akt signaling plays a vital role in the regulation of cell growth, metabolism, proliferation, glucose homeostasis, and vesicle trafficking [41]. One of the key mechanisms observed in the tissues impacted by type 2 diabetes is that PI3K/Akt-mediated NF-*κ*B signaling might be a mechanism for the treatment of DN [16]. The status of renal cortical PI3K/Akt signaling pathways was activated and higher in diabetic mice in the early phase of diabetic nephropathy [42]. Of note, other than stimulating TGF-*β*1 secretion, there is also evidence indicating that IL-1*β* can activate PI3K/Akt signaling [43], thereby enhancing renal fibrosis [41]. A previous study proposed a role for PI3K/Akt as a possible regulator of cell survival after mangiferin exposure [44]. Therefore, due to the potential DN related pathways, the mechanism of the PI3K/Akt signaling pathway in mangiferin inhibiting renal interstitial fibrosis was further explored. In this study, STZ prominently increased the expressions of phosphorylated PI3K and Akt, which are consistent with the conclusions reported in previous reports [16, 42]. In addition, the administration of mangiferin significantly decreased the expression of phosphorylated PI3K and Akt in the fibrotic kidneys. A similar report also revealed that TB-II, a main ingredient of A. asphodeloides Bunge, notably improved cell viability by decreasing p-PI3K and p-Akt [28]. These findings supported that PI3K/Akt signaling may be involved in renal fibrosis induced by STZ and intervened by mangiferin.

Furthermore, a changing situation of PTEN was observed in the injured kidneys of diabetic mice. The tumor suppressor PTEN was originally identified as a negative regulator of the PI3K signaling; it can inhibit the activation of Akt [45]. The current study indicated that renal fibrosis of model mice evidenced by morphological and molecules indices in kidney was related with the downregulation of PTEN protein; however, mangiferin treatment effectively reversed these alterations. Moreover, due to the critical role of PTEN in DN, with the use of PTEN inhibitor BpV, the development of renal interstitial fibrosis was aggravated in mice models through the increased Col I, FN, *α*-SMA, and TGF-*β*1, as well as the elevated PI3K/AKT signaling pathway. Furthermore, the beneficial effect of mangiferin on renal interstitial fibrosis was relieved when BpV was presented, but not greater than without BpV. The loss or impairment of PTEN results in an antidiabetic impact, which led to the suggestion that PTEN could be an important target for drugs against type II diabetes. In this study, when the PTEN inhibitor was used, the protein expressions of TGF-*β*1, p-PI3K, and p-AKT were higher. But the expressions of PTEN change little without significance, which means the mangiferin exhibits an antifibrosis effect through regulating the activation of PTEN. The PTEN inhibitor blocks the activating effect of mangiferin. These findings demonstrated that mangiferin prevented the progression of STZ-induced DN pathology, and upregulation of PTEN may be one of the important reasons. Many animal studies and further clinical data have revealed that reduced PI3K activity can play a role in insulin sensitivity and type II diabetes [45]. Thus, one of the underlying mechanisms by which mangiferin attenuates DN may be to increase the insulin sensitivity by inhibiting the PI3K/Akt signaling pathway. These findings indicated that the PI3K/Akt signaling pathway exhibited a critical role in renal interstitial fibrosis, and alternative approaches which inhibited the PI3K/Akt signaling pathway and activated PTEN dependent activity might be a potential method of protecting against renal injury.

## 5. Conclusions

This study indicated that mangiferin, as an effective antifibrogenic agent, regulated the PTEN/PI3K/Akt pathway, thereby inhibiting the renal interstitial fibrosis in DN by reducing the TGF-*β*1-induced elevation of Col I, FN, and *α*-SMA. Our findings yield novel insights into the molecular mechanisms of mangiferin in renal interstitial fibrosis and provide new therapeutic approaches for chronic fibrotic kidney disease. Mangiferin might be used as a potential adjuvant for preventing and treating DN. However, this study has some limitations. Although the expression patterns of several proteins were shown, they were not the complete molecular mechanisms involved in DN; more factors need further clarity.

## Data Availability

The data used to support the findings of this study “Mangiferin Alleviates Renal Interstitial Fibrosis in Streptozotocin-Induced Diabetic Mice through Regulating the PTEN/PI3K/Akt Signaling Pathway” are included within the article and available from the corresponding author upon request.
